# Qi-Fu-Yin ameliorates physiological frailty in male 5xFAD mice through remodeling the gut microbiota and modulating the cerebral cortex metabolism

**DOI:** 10.3389/fnagi.2025.1622286

**Published:** 2025-12-16

**Authors:** Yitong Xiao, He Li, Xinyuan Han, Yixiao Liu, Jiayu Sun, Chenxi Sun, Yichen Wang, Tianyuan Ye, Xiaorui Cheng

**Affiliations:** 1 College of Traditional Chinese Medicine, Shandong University of Traditional Chinese Medicine, Jinan, China; 2 Innovative Institute of Chinese Medicine and Pharmacy, Shandong University of Traditional Chinese Medicine, Jinan, China; 3 College of Medicine, Shandong University of Traditional Chinese Medicine, Jinan, China

**Keywords:** Alzheimer’s disease, Qi-Fu-Yin, physiological frailty, 16S rRNA sequencing, metabolomics

## Abstract

**Introduction:**

Alzheimer’s disease (AD) is a neurodegenerative disease that can only be managed rather than cured, bringing a substantial burden to society. Frailty and cognition are intertwined in a cycle of decline, affecting the prognosis of AD. Qi-Fu-Yin (QFY) is a classic prescription in traditional Chinese medicine for dementia. While most studies have focused on cognitive impairment, research on physiological frailty remains relatively scarce in AD, especially in 5xFAD mice. We aimed to investigate the impacts of QFY on the physiological frailty of male 5xFAD mice.

**Methods:**

Male 5xFAD mice received QFY, followed by grip strength test, rotarod test, grading score of frailty, lipofuscin staining, SA-β-gal and Aβ co-staining. The metabolite alteration and the intestinal flora composition were analyzed by non-targeted metabolomics and 16S rRNA sequencing. Moreover, Spearman’s correlation analysis was used to integrate behavioral results, differentially expressed metabolites, and altered bacterial genera.

**Results:**

We discovered that QFY improved grip strength, riding time, score of frailty, lipofuscin deposition, SA-β-gal, and Aβ in male 5xFAD mice. The results of untargeted metabolomics showed that metabolites such as proline, PS (18:1/18:0), and PFSA-CI were downregulated in the male 5xFAD mice compared with C57BJ/6JXSJL mice, while PE (18:1/18:1) was upregulated. QFY treatment reversed these changes, restoring metabolite levels toward those of C57BJ/6JXSJL mice. Arginine and proline metabolism, alanine, aspartate and glutamate metabolism, and butyrate metabolism were filtered out as the important metabolic pathways between the C57BJ/6JXSJL mice and the male 5xFAD mice, as well as between the 5xFAD mice and the 5xFAD mice with QFY treatment. Moreover, Ruminococcaceae, Subdoligranulum, Bacteroides, Alistipes, Rikenellaceae_RC9_gut_group, and Odoribacter, which were lower in male 5xFAD mice, were improved after QFY intervention.

**Discussion:**

The differential intestinal flora might improve the metabolism of brain tissue as well as muscle strength and coordination through Short-chain fatty acids (SCFAs). The differential metabolites caused by QFY intervention also have an improving effect on physiological frailty. We suggest that QFY exerts protective impacts against the physiological frailty in AD by adjusting the muscle-gut-brain axis.

## 1 Introduction

Frailty, a syndrome that can be identified in a clinical setting, is related to the aging of various physiological systems (Gómez-Gómez and Zapico, 2019). Distinguished by the decline of muscle function, frailty grows more common with age, laying a large burden on patients and caregivers (Deng et al., 2023). It exacerbates the continuous decline of cognitive function and raises the susceptibility to Alzheimer’s disease (AD) (Jang et al., 2023; Chung et al., 2022). Frailty and cognition are intertwined in a cycle of decline, increasing the risk of adverse clinical outcomes (Robertson et al., 2013). Focusing on and timely intervening in the physiological frailty of people with AD may become a novel strategy to delay AD progression and improve their quality of life. At present, widely recommended physical and psychological strategies include exercise, calorie restriction (such as through a healthy diet), anti-aging neuro-protection, and anti-inflammation therapies (Zhou, 2019). They help to maintain a good metabolic profile and regulate the intestinal flora, which plays a significant role in improving the quality of life during aging.

Numerous studies have verified the crucial effect that the gut-brain axis has on frailty (Ticinesi et al., 2018). Muscle-gut-brain axis, which serves as an expansion of the gut-brain axis, has been steadily growing in popularity in recent years. This axis highlights associations among the aging process, gut dysbiosis, metabolic changes in brain tissue, and modifications to muscle functionality. Signals released from the gut can affect muscle function by regulating inflammatory processes and modulating insulin sensitivity (Giron et al., 2022). Also, muscle movement in physical exercise can transform the constitution of the gut microbiota (Cammisuli et al., 2022). The brain controls muscle activities through a complex neural network, sending out electrical and chemical signals to directly command muscles. Currently, many studies consider the muscle as an endocrine organ. The neurotrophic factors and myokines released from the muscle can regulate synapses in the brain, thereby controlling brain functions, and acting as mediators for the advantageous effects that physical exercise has on the brain (Isaac et al., 2021; Sui et al., 2020).

In recent years, metabolomics has emerged as a prominent approach in AD research. It has recently been reported that significantly higher concentrations of 15 metabolites, including 12 phosphatidylcholines (PCs) and 3 sphingomyelins (SMs), were found in people with early probable AD compared to controls based on metabolomics (Zhou, 2021).

Qi-Fu-Yin (QFY) was first recorded in the Ming Dynasty and is one of the classic prescriptions in traditional Chinese medicine (TCM) for dementia. The formula consists of seven traditional Chinese medicinal herbs: *Panax ginseng*, the most important ingredient in QFY, is considered in TCM to tonify primordial qi—an effect that helps maintain the body’s vital activities and regulate the function of internal organs to enhance resistance to aging-related disorders (e.g., muscle degeneration and memory decline). Other ingredients include: Rehmannia Glutinosa and Angelicae Sinensis (thought to nourish blood and promote blood circulation); fried Atractylodis Macrocephalae, Semen Ziziphi Spinosae, and prepared Polygala (thought to promote digestion and absorption, tranquillize, and facilitate sleep); and roasted Glycyrrhiza (thought to harmonize all medicinal ingredients). Experimental pharmacological studies have found that its possible anti-AD mechanism is to inhibit neuronal apoptosis, reduce inflammation in the brain, ameliorate oxidative stress, and regulate abnormal immunity (Lei et al., 2023; Wang S. et al., 2024; Yang X. et al., 2023).

The 5xFAD mouse is a widely used model in AD research because it exhibits early-onset symptoms characteristic of late-stage AD in humans (Bilkei-Gorzo, 2014). These mice are characterized by the overexpression of five mutant human genes associated with early-onset familial AD: three encoding amyloid precursor protein (APP) and two encoding presenilin 1 (PSEN1).

Muscle frailty is a crucial component of physiological frailty. Studies have shown that compared with females, males have a higher prevalence of sarcopenia, a more rapid disease progression, and a stronger association with health risks, with the decline in muscle function preceding the loss of muscle mass (Kerr et al., 2024). Meanwhile, the pathological mechanisms of sarcopenia in males are fundamentally different from those in females, and universal intervention strategies targeting “inhibition of canonical atrogenes” are not applicable to males (Owen and Fry, 2024; Edström et al., 2006). Furthermore, it has been suggested that the risk of AD can be adjusted following the emergence of mild cognitive impairment, and the count of dementia cases that could potentially be prevented might be greater among males compared to females (Martin et al., 2025). Therefore, greater attention should be paid to males regarding physiological frailty in AD.

While most existing studies have focused on cognitive impairment, research on physiological frailty remains relatively scarce in AD, especially in 5xFAD mice (Todorovic et al., 2020). Given that physiological frailty significantly impacts the quality of life in people with AD and that frailty assessment can identify individuals prone to severe AD progression while measuring outcomes of existing and putative AD therapies, we aimed to investigate the underexplored territory of physiological frailty in AD and the impacts of QFY on the physiological frailty of male 5xFAD mice. In addition, the non-targeted metabolomics and 16S rRNA sequencing were applied to further investigate the latent therapeutic mechanism of QFY.

## 2 Materials and methods

### 2.1 Preparation of QFY

QFY was prepared by Lunan Pharmaceutical Group Co., Ltd. (Shandong, China, Batch number: 2209001). Major constituents were as follows: *Panax ginseng* 3.0 kg, cooked Rehmannia Glutinosa 4.50 kg, Angelicae Sinensis 4.50 kg, fried Atractylodis Macrocephalae Rhizoma 2.50 kg, Semen Ziziphi Spinosae 3.0 kg, prepared Polygala 2.50 kg, roasted Glycyrrhiza 1.50 kg.

For the above seven ingredients, Ginseng was refluxed with 60% ethanol twice, each time lasting 1.5 h. Then it was filtered, and the drug residues were reserved for later use. The filtrate was subjected to ethanol recovery and concentrated until the relative density reached 1.03–1.10 (at 60 °C), and then reserved for subsequent steps.

Radix Angelicae Sinensis and fried Atractylodis Macrocephalae Rhizoma were used to extract volatile oil by the water distillation method. The aqueous solution after distillation was collected in another container, and the drug residues were reserved. The volatile oil ethanol solution was included with beta-cyclodextrin, dried, and pulverized for reserve.

The above three kinds of drug residues were combined with the other four ingredients, including cooked Rehmannia glutinosa, etc., and then decocted with water twice, with each time lasting for 2 h. After filtration, the resulting liquid was combined with the concentrated solution of Ginseng and the liquid after oil extraction, and then concentrated to obtain a clear paste. It was then allowed to stand still and centrifuged, followed by further concentration to obtain an extract with a relative density of 1.22–1.28 (at 60 °C). Subsequently, it was dried and pulverized. Finally, the beta-cyclodextrin inclusion complex was added and mixed evenly.

### 2.2 Animals and treatment

The 5xFAD transgenic mice were acquired from Jackson Laboratories (Stock No.034840, Bar Harbor, ME, USA) and bred to ensure the continuation of the colony. PCR was used to confirm the genotypes of animals: Hemizygous 5xFAD mice and their wild-type littermates were employed for the experiment. The wild-type control group consisted of C57BL/6JXSJL mice without the 5xFAD transgene, genetically matched to the experimental group to minimize background-related variability. The mice were housed in the SPF barrier environment with a 12-h light/dark cycle, a temperature of 22 ± 2 °C, and a relative humidity of 55 ± 10%, with free access to food and water.

**Figure 1:**
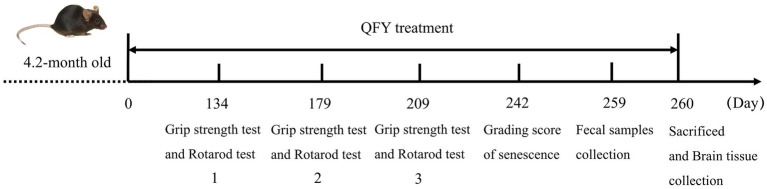
The workflow of animals and drug administration.

When male mice were 4.2 months old, they were randomly split into three groups (*n* = 21–27): (1) QFY group, comprising 5xFAD mice that were intervened with QFY (2.1 g/kg/d) via intragastric administration once a day; (2) wild type(WT) group, comprising C57/B6XSJL mice that were intervened with equivalent volume of water; (3) 5xFAD group, comprising 5xFAD mice that were intervened with equivalent volume of water. The behavioral experiments were employed from 134 days to 209 days of QFY feeding (Figure 1). After that, the brain and feces were collected for lipofuscin staining, SA-β-gal and Aβ co-staining, metabolomic profiling, and 16S rRNA sequencing.

### 2.3 Behavioral tests

#### 2.3.1 Grip strength test

The grip strength measuring device was positioned flat, and the mouse was placed on the grip strength bar. Once the mouse grasped the bar firmly, a uniform backward pull was exerted until it released the plate, while the instrument automatically recorded the mouse’s maximum grip strength. The above steps were repeated three times, and the average of these three measurements was regarded as the mouse’s grip strength value.

#### 2.3.2 Rotarod test

Mice were put on the rotating track of the rotarod instrument and allowed 30 s to adapt to the environment. Then the rotarod was initiated, with the rotation speed set to accelerate uniformly to 40 r/min within 120 s and maintained at 40 r/min for 180 s until the training ended or the mice fell off. Twenty-four hours later, the formal experiment was conducted with the same parameters as those in the training. The duration of the mice staying on the rotarod was recorded. The trial was taken three times with a 30-min gap separating each instance, and the average value of these three experiments was regarded as the rotarod value of the mice.

#### 2.3.3 Degree of frailty

The frailty score table for mice was formulated based on the frailty scoring criteria developed by Professor Toshio Takeda and Masanori Hosokawa from Kyoto University in Japan (Hosokawa et al., 1984). The physiological frailty of mice was scored in detail using four key indicators: behavioral responses, skin and hair condition, ocular status, and spinal morphology (Supplementary Table 1). One person placed the mice on the observation platform and applied corresponding stimuli following the scoring indicators, while three people, who were blind to the mice’s group assignment and serial numbers during the experiment, observed and scored the mice. The average value of the scores given by the three people was taken as the score of the frailty of each mouse.

### 2.4 Collection and pre-treatment of mouse samples

Six mice were randomly selected from each of the control, model, and QFY groups. Feces were collected from all mice 1 day before sacrifice and stored at −80 °C until testing. The mice were sacrificed by decapitation, and the whole brains were isolated. The whole brains were rinsed thoroughly with water and then split into two halves along the gap between the left and right cerebral hemispheres. Three of the left hemispheres were immersed in alcohol and xylene sequentially. They were then immersed in a 60 °C wax solution for 3 h, transferred to an embedding cassette, and stored for later use once the wax solution solidified. Three of the left hemispheres were fixed in 4% paraformaldehyde, followed by gradient sedimentation in 15 and 30% sucrose solutions sequentially. They were then embedded in optimal cutting temperature compound (OTC) and stored in a −80 °C refrigerator for pathological staining. The cerebral cortex tissue of the right hemisphere was dissected, placed into a centrifuge tube, snap-frozen in liquid nitrogen, and then stored in a −80 °C refrigerator for subsequent untargeted metabolomics analysis.

### 2.5 Lipofuscin staining

The left hemisphere samples were cut into 4 μm sections and conventionally dewaxed and hydrated. The ferric chloride solution, potassium ferricyanide solution, and distilled water were mixed at a ratio of 7.5:1:1.5 (Gefanbio, Shanghai, China), and the samples were treated for 8 min. The neutral red staining solution (Gefanbio, Shanghai, China) was used to cover the samples for 8 min after washing with water. Samples were washed with water, dehydrated in a hot air circulating oven, cleared with xylene, and mounted with resin. Results were observed using a light microscope.

### 2.6 SA-β-gal and Aβ co-staining

Three left hemisphere samples were removed from the −80 °C refrigerator, equilibrated to −20 °C, and cut into 40 μm sections. β-gal Fixative (Solarbio, Beijing, China) was added to cover samples for 15 min after washing with PBS. SA-β-gal dyeing working solution (Solarbio, Beijing, China) was added and maintained at 37 °C overnight after removal of β-gal Fixative and washing with PBS three times. 70% ethanol was used to dissolve crystals, and xylene was added to make the samples transparent. Results were observed using a light microscope.

Samples were hydrated with ethanol and washed with PBST, and immersed in the citrate buffer solution at 99 °C for 25 min. The blocking solution (Absin, Shanghai, China) was introduced for 10 min after washing with PBST three times. Anti-Aβ1–42 was incubated (1:400, Abcam, Cambridge, UK) at 4 °C for the entire night after washing with PBST three times. Enhanced enzyme-labeled IgG polymer (Absin, Shanghai, China) was incubated for 35 min under dark conditions after washing with PBST three times. The DAB kit (Absin, Shanghai, China) was used for color development for 15 min after washing with PBST. Samples were dehydrated and cleared with alcohol and xylene, and sealed with neutral gum.

Results were observed using a light microscope. One section per sample was selected for imaging: hemibrain sections were captured at 2 × magnification, while hippocampal and cortical regions of interest were imaged at 40 × magnification in single-plane mode. Quantification was performed using ImageJ.^1^ Briefly, color images were first split into individual channels, and the suitable channel was selected. A consistent threshold was applied across all samples to distinguish positive staining signals from background. Then the positive area fraction was calculated.

### 2.7 Metabolomic profiling

The prepared cerebral cortex tissue of the right hemisphere was ground in an Eppendorf tube containing a tungsten bead for 1 min at 65 Hz using a Grinding Mill. Metabolites were extracted via a pre-chilled mixture of methanol, acetonitrile, and water (v/v/v, 2:2:1). The mixture was then ultrasonicated and centrifuged. The supernatants were retrieved and concentrated to dryness under a vacuum environment, then dissolved in 50% acetonitrile. This was transferred into HPLC vials after being filtered with 0.22 μm cellulose acetate for analysis.

A UPLC-ESI-Q-Orbitrap-MS system (UHPLC, Shimadzu Nexera X2 LC-30 AD, Shimadzu, Japan) and Q-Exactive Plus (Thermo Scientific, San Jose, USA) were used to analyze the metabolomic profiling. LC separation was performed through the ACQUITY UPLC® HSS T3 column (2.1 × 100 mm, 1.8 μm) (Waters, Milford, MA, USA). SIMCAP software (Version 14.0, Umetrics, Umeå, Sweden) was utilized to conduct all multivariate data analyses and build models. Pathway analysis was carried out on the differential metabolite data using the MetaboAnalyst database^2^ and the KEGG database.^3^

### 2.8 Data analysis of fecal 16S rRNA sequencing

The extraction of total genomic DNA samples in feces was accomplished with the use of the OMEGA Soil DNA Kit (M5635-02) (Omega Bio-Tek, Norcross, GA, USA). After detecting the DNA purity, PCR amplification of the 16S rRNA gene at the V3-V4 region was conducted using the forward primer 338F (5′-ACTCCTACGGGAGGCAGCA-3′) and the reverse primer 806R (5′-GGACTACHVGGGTWTCTAAT-3′).

QIIME2 and R packages (v3.2.0) were used following the official tutorials^4^ for sequence data modification and analyses. Sequences were quality filtered, denoised, merged, and chimera removed through the DADA2 plugin. Mafft was employed to align non-singleton amplicon sequence variants (ASVs). Alpha-diversity metrics (Chao1, Shannon) were visualized as box plots, and beta-diversity metrics were visualized via principal coordinate analysis (PCoA) using the diversity plugin. LEfSe (Linear discriminant analysis effect size) was performed to detect differentially abundant taxa across groups with the default parameters.

### 2.9 Correlation analysis among behavioral data, gut microbiota, and metabolites

Spearman’s correlation analysis was performed to analyze the relationship between behavioral data, differential metabolites, and differential gut microbiota in the control, 5xFAD, and QFY groups.

### 2.10 Statistical analysis

Data were presented as mean ± SEM and analyzed by GraphPad Prism 8. Normality was tested using the Shapiro–Wilk test. Results were analyzed using the unpaired Student’s *t*-test. Repeated tasks were assessed individually at each time point only. Statistical significance was considered at *p* < 0.05.

## 3 Results

### 3.1 QFY improved physiological frailty in male 5xFAD mice

**Figure 2:**
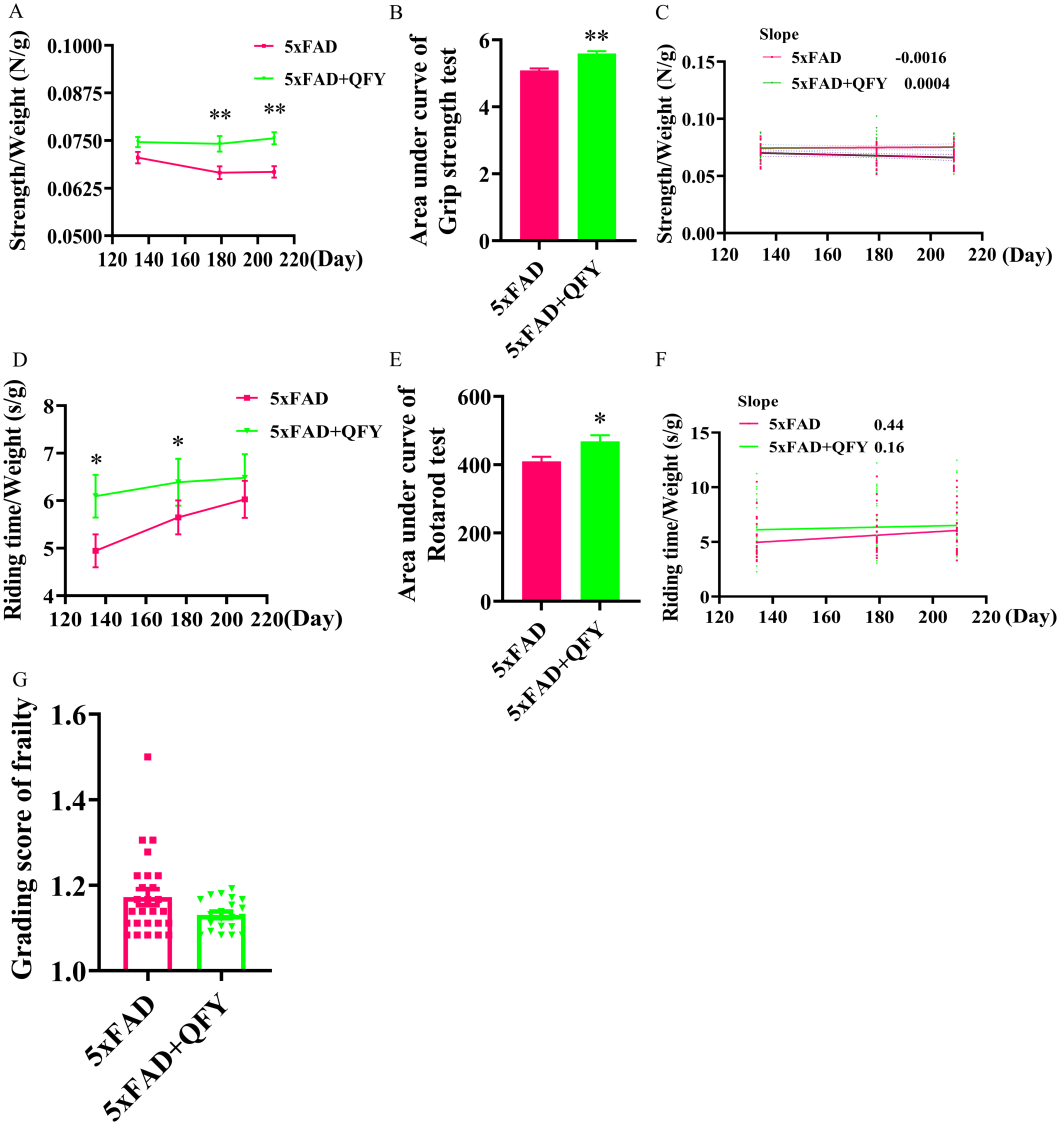
Effects of QFY on physiological frailty of 5xFAD mice. Line plot **(A)** area under line plot **(B)** and scatter plot with linear fit **(C)** of grip strength test. Line plot **(D)** area under line plot **(E)** and scatter plot with linear fit **(F)** of rotarod test. **(G)** Grading score of frailty. In figures **A**, **B**, **D**, **E**, and **G**, data are expressed as the mean ± SEM, *n* = 21–27; ^*^p < 0.05, ^**^p < 0.01 *vs* 5xFAD mice. Statistical differences are assessed by the Student’s *t*-test.

On the 134th, 179th, and 209th days of QFY intervention, grip strength test and rotarod test were conducted to evaluate the forelimb strength and motor coordination ability of male mice (Figure 1). The grip strength/weight was significantly greater in the QFY group relative to the model group at day 179 (*p* < 0.01) and day 209 (*p* < 0.01) (Figure 2A). The area under the curve (AUC) of the grip strength/weight was significantly greater in the QFY group relative to the model group (*p* < 0.01) (Figure 2B). Scatter plot with linear fit showed greater value of slope in the QFY group relative to the model group, indicating that the grip strength/weight decreased more slowly with age in 5xFAD mice with QFY intervention, but there were no significant differences (Figure 2C).

The riding time/weight significantly enhanced in the QFY group relative to the model group at day 134 (*p* < 0.05) and day 180 (*p* < 0.05) (Figure 2D). Riding time was normalized to body weight (expressed as s/g) to account for the well-documented negative correlation between body weight and rotarod performance in C57BL/6 J mice (McFadyen et al., 2003). The AUC of the riding time/weight was significantly greater in the QFY group relative to the model group (*p* < 0.05) (Figure 2E). Scatter plot with linear fit showed a smaller value of slope in the QFY group relative to the model group, indicating that QFY might have a stronger enhancing effect on motor coordination ability in the early stage of 5xFAD mice, but there were no significant differences (Figure 2F).

After QFY treatment for 242 days, a grading score of frailty was employed. The frailty score of the QFY group was lower than that of the 5xFAD group, but there were no significant differences (Figure 2G).

These results indicated that QFY could delay physiological frailty in male 5xFAD mice.

### 3.2 QFY slowed the brain aging and alleviated typical pathological features of AD in 5xFAD mice

**Figure 3:**
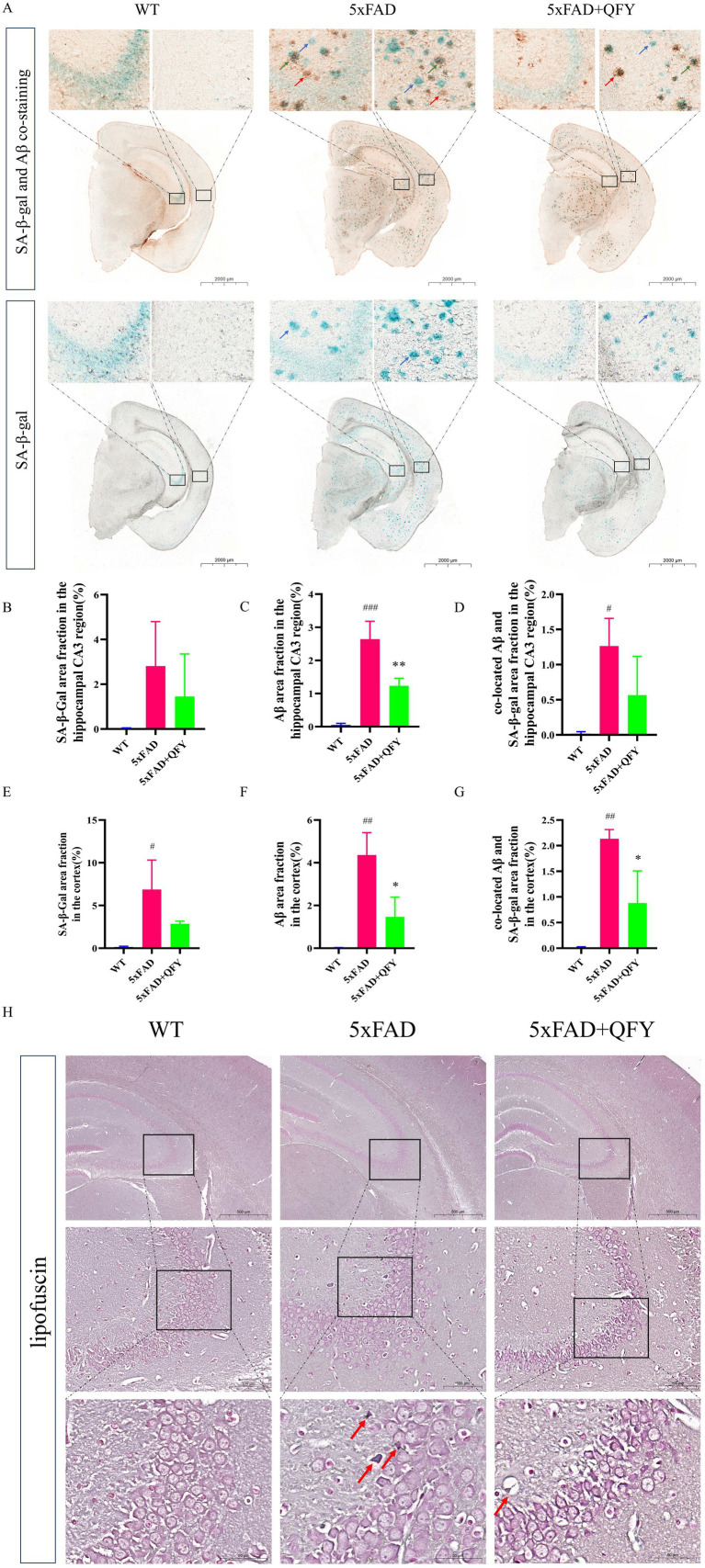
Effects of QFY on the deposition of lipofuscin, SA-β-gal, and Aβ in the brain of 5xFAD mice. **(A)** Representative images of SA-β-gal and Aβ co-staining, and SA-β-gal staining. The red arrow points to amyloid deposition, the blue arrow points to SA-β-gal-positive cells, and the green arrow points to co-located amyloid deposition and SA-β-gal-positive cells in the cortex and hippocampus. **(B–D)** Quantification of SA-β-gal, Aβ, as well as co-located Aβ and SA-β-gal area fraction in the hippocampal CA3 subregion. **(E–G)** Quantification of SA-β-gal, Aβ, as well as co-located Aβ and SA-β-gal area fraction in the cerebral cortex. **(H)** Representative images of lipofuscin staining. The red arrow points to lipofuscin. In figures **B**–**G**, data are expressed as the mean ± SEM, *n* = 3; *^#^p* < 0.05, ^##^*p* < 0.01, ^###^*p* < 0.001 *vs* WT mice; *p < 0.05, **p < 0.01 *vs* 5xFAD mice. Statistical differences were assessed by analysis of variance (ANOVA) followed by Tukey’s multiple comparison test.

We detected the deposition of lipofuscin, SA-β-gal, and Aβ in the brains of mice (Figure 3A). Compared with WT mice, SA-β-gal-positive cells, amyloid deposition, as well as co-located amyloid deposition and SA-β-gal-positive cells were observed in both the hippocampus (using the CA3 region as an example) and cortexes of 5xFAD mice. These positive signals were less abundant in 5xFAD mice with QFY intervention, and statistically significantly lower levels were observed in three metrics: the Aβ-positive area in the hippocampal CA3 region, the Aβ-positive area in the cortex, and the Aβ/SA-β-gal co-localized area in the cortex (Figures 3B–G).

Studies have shown that the CA3 subregion of the hippocampus exhibits a higher accumulation of lipofuscin compared to other hippocampal subregions, and this accumulation is directly associated with neuronal functional decline (Sharma et al., 1993). Therefore, the CA3 subregion was selected to demonstrate the lipofuscin deposition. Qualitative observations suggest blue lipofuscin deposition appeared in the CA3 regions of the hippocampus of the 5xFAD mice compared with the WT mice. Additionally, a perceptible reduction in lipofuscin staining intensity was observed in the 5xFAD mice with QFY intervention relative to the 5xFAD mice, but this trend of less lipofuscin deposition remains preliminary and requires confirmation through quantitative analyses (Figure 3H). These results implied that QFY also slowed brain aging and alleviated the typical pathological features of AD in male 5xFAD mice.

### 3.3 QFY altered the cerebral cortex metabolic profiles of male 5xFAD mice

**Figure 4:**
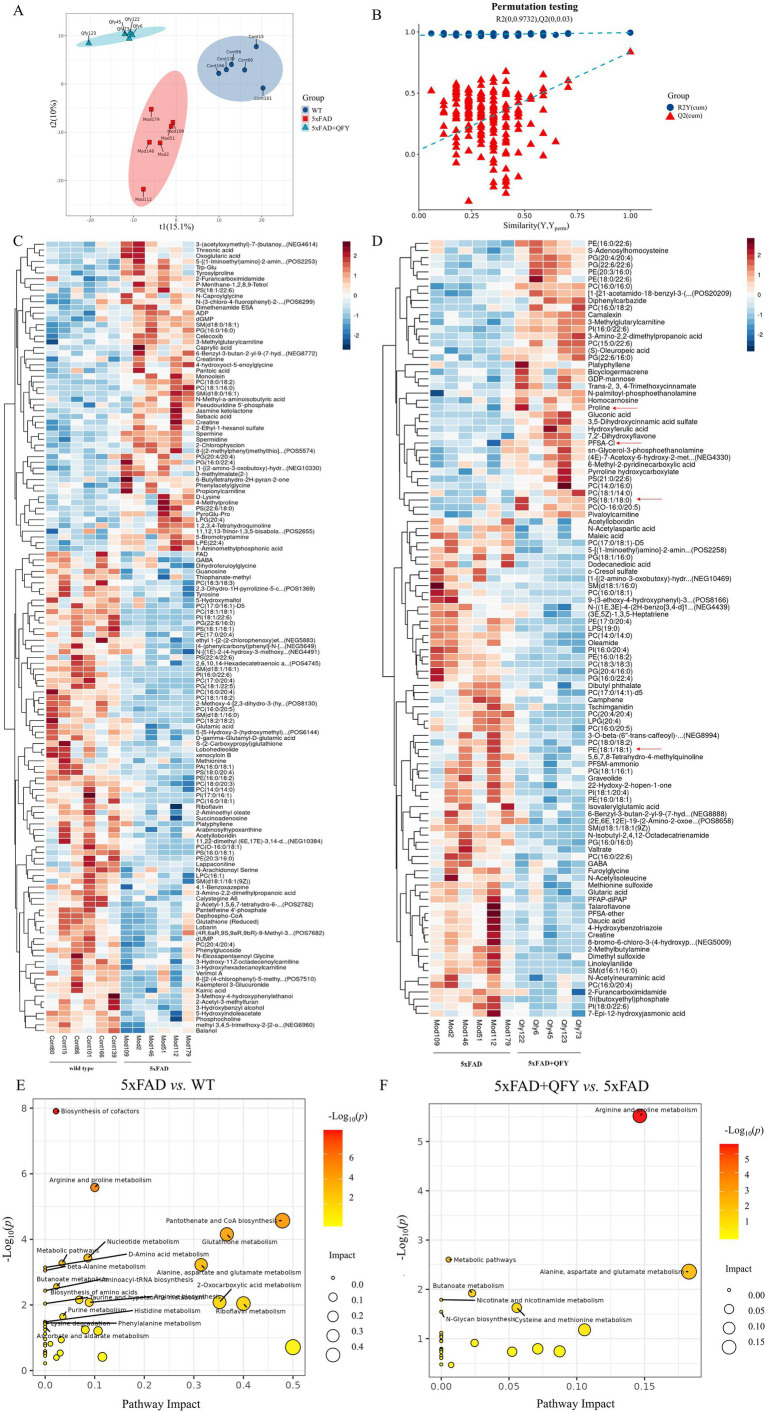
The cerebral cortex metabolic profile of mice following intervention with QFY. **(A,B)** Scores plot of PLS-DA and the corresponding coefficient of loading plot. **(C,D)** Heatmaps of hierarchical clustering analysis of metabolites between WT mice and 5xFAD mice, and between 5xFAD mice and 5xFAD + QFY mice. **(E,F)** Metabolic pathway analysis between WT mice and 5xFAD mice, and between 5xFAD mice and 5xFAD + QFY mice. *n* = 5–6.

The alterations in metabolites within the cerebral cortex were investigated using untargeted metabolomics. PLS-DA revealed distinct group separations among the three groups (Figure 4A). The overfitting in the PLS-DA model was evaluated by 200 repetitions of random permutation testing (RPT). The Q2 value was less than the R2 value, indicating the robustness of the PLS-DA model (Figure 4B).

Metabolites exhibiting significant differences were identified using a criterion VIP > 1 in the PLS-DA model and a significance level of *p* < 0.05. Most of the differential metabolites between the WT mice and the 5xFAD mice, as well as between the 5xFAD mice and the 5xFAD mice with QFY treatment, were lipids and lipid-like molecules. Hierarchical clustering analysis was used to screen significantly different metabolites between the WT mice and the 5xFAD mice, as well as between the 5xFAD mice and the 5xFAD mice with QFY treatment (Figures 4C,D). Several metabolites including proline (*p* < 0.05), PS (18:1/18:0) (*p* < 0.05), and PFSA-CI (*p* < 0.05) were downregulated in the 5xFAD mice relative to WT mice, while PE (18:1/18:1) (*p* < 0.05) was upregulated in the 5xFAD mice relative to WT mice, and these were all reversed by the intervention of QFY (Supplementary Table 2).

In the pathway enrichment analysis of differential metabolites, arginine and proline metabolism, alanine, aspartate and glutamate metabolism, and butyrate metabolism were screened as key metabolic pathways between WT mice and 5xFAD mice, as well as between 5xFAD mice and 5xFAD mice with QFY treatment (Figures 4E,F).

### 3.4 QFY regulated the gut microbiota composition of male 5xFAD mice

**Figure 5:**
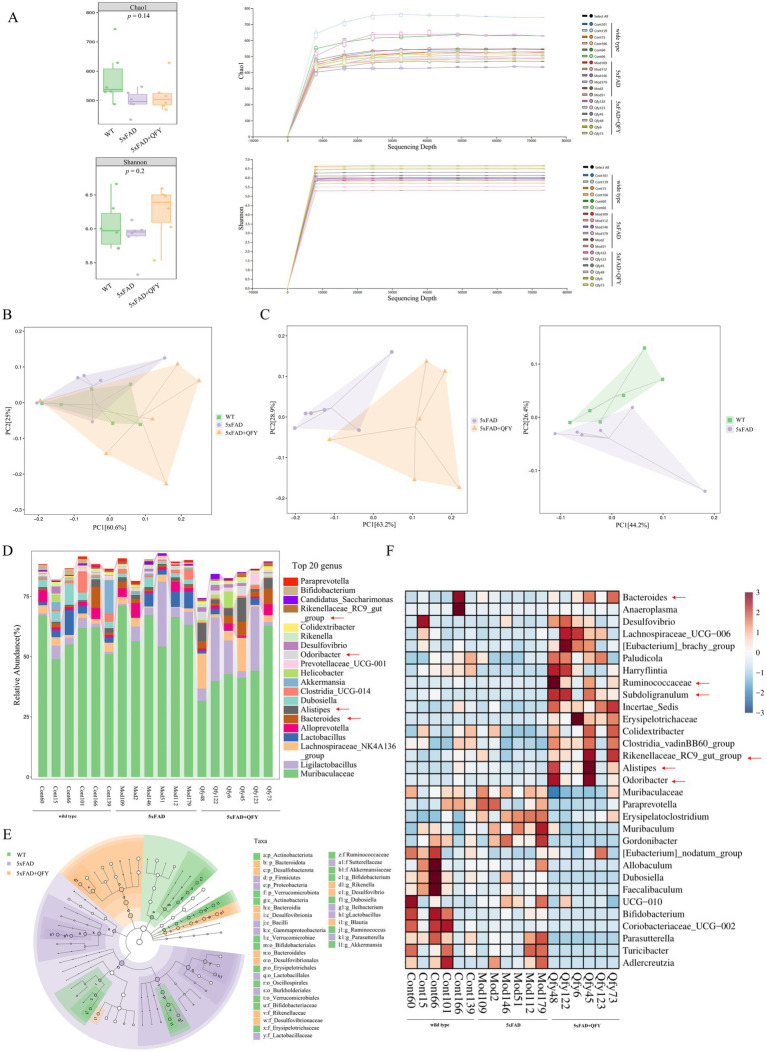
Effects of QFY on the composition of gut microbiota in 5xFAD mice. **(A)** *α*-diversity analysis based on the Chao and Shannon index and Rarefaction curve. **(B)** PCoA plots of β-diversity at the order level. **(C)** PCoA plots of β-diversity at the genus level. **(D)** Relative abundance of species at the genus level. **(E)** LEfSe comparison of gut microbiota. **(F)** The heatmap of species composition at the genus level. In figure **A**, data are expressed as the mean ± SEM, *n* = 6.

16S rRNA sequencing was conducted to identify the alterations in the gut microbiota of 5xFAD mice following QFY treatment. A total of 1,795,371 valid sequences and 2,804 ASVs were obtained from the sequencing of 18 samples, and the lengths of the sequences mostly fell within the range of 404–432 bp. The Shannon (diversity index) and Chao indices (richness index) were applied to analyze the *α*-diversity data, which showed no significant differences among the three groups. With an increase in sampled sequence numbers, the Shannon and Chao curves flattened, demonstrating that the amount of sequencing data was reasonable (Figure 5A). Principal co-ordinates analysis (PCoA) demonstrated a similar trend and closer proximity between the WT mice and the 5xFAD mice with QFY treatment (Figure 5B), while significant variations of gut microbiota were found in three groups at the genus level (Figure 5C). These findings suggested that QFY induced modifications in the overall structure of gut microbiota in 5xFAD mice to a certain extent.

**Figure 6:**
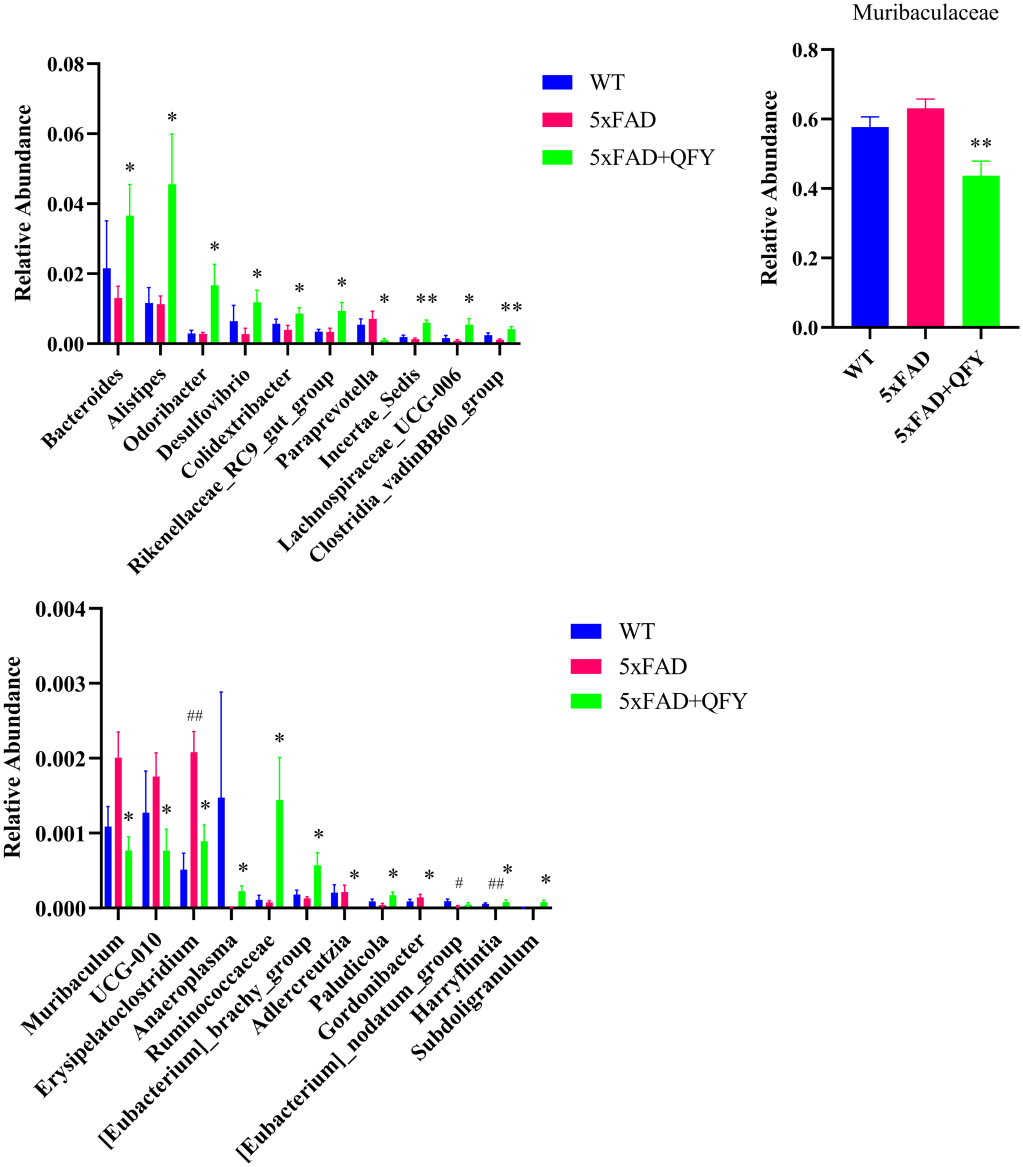
Gut microbiota exhibiting significant differences: either between WT mice and 5xFAD mice or between 5xFAD mice and 5xFAD + QFY mice, and showing a co-directional change trend between WT mice and 5xFAD + QFY mice. Data are expressed as the mean ± SEM, *n* = 6; *^#^p* < 0.05, ^##^*p* < 0.01, *vs* WT mice; *p < 0.05, **p < 0.01 *vs* 5xFAD mice. Statistical differences were assessed by the Student’s *t*-test.

At the genus level, 221 intestinal bacteria were identified among the three groups. The relative abundance analysis of the three groups showed that the main dominant bacterial group was *Muribaculaceae* (Figure 5D). Moreover, the LEfSe method was applied to single out the remarkably distinct specific bacteria among the three groups, based on the screening criteria of LDA > 2 and *p* < 0.05 (Figure 5E). 38 specific key genera were found in total, with 16 enriched in control, 11 in model, and 11 in QFY. These subtypes significantly contributed to the discrimination among the three groups. The abundance of 31 intestinal bacterial genera was significantly different between WT mice and 5xFAD mice, as well as between 5xFAD mice and 5xFAD mice with QFY treatment (Figure 5F). Remarkably, the abundance of gut microbiota taxa, such as *Ruminococcaceae* (*p* < 0.05), *Subdoligranulum* (*p* < 0.05), *Bacteroides* (*p* < 0.05), *Alistipes* (*p* < 0.05), *Rikenellaceae_RC9_gut_group* (*p* < 0.05), *Odoribacter* (*p* < 0.05), *Desulfovibrio* (*p* < 0.05), *Colidextribacter* (*p* < 0.05), *Incertae_Sedis* (*p* < 0.01), *Lachnospiraceae_UCG-006* (*p* < 0.05), *Clostridia_vadinBB60_group* (*p* < 0.01), *Anaeroplasma* (*p* < 0.05), *[Eubacterium]_brachy_group* (*p* < 0.05), *Paludicola* (*p* < 0.05), *[Eubacterium]_nodatum_group* (*p* < 0.05), and *Harryflintia* (*p* < 0.05), was lower in 5xFAD mice relative to WT mice, while it was dramatically higher in the QFY group compared to the 5xFAD group. The abundance of *Muribaculaceae* (*p* < 0.01), *Paraprevotella* (*p* < 0.05), *Muribaculum* (*p* < 0.05)*, UCG-010* (*p* < 0.05)*, Erysipelatoclostridium* (*p* < 0.05), *Adlercreutzia* (*p* < 0.05), and *Gordonibacter* (*p* < 0.05) was higher in 5xFAD mice relative to WT mice, while it was lower in the QFY group compared to the 5xFAD group (Figure 6). These results indicated that QFY modulated the composition of gut microbiota in male 5xFAD mice.

### 3.5 Correlation analysis of behavioral data, metabolomics, and gut microbiota

**Figure 7:**
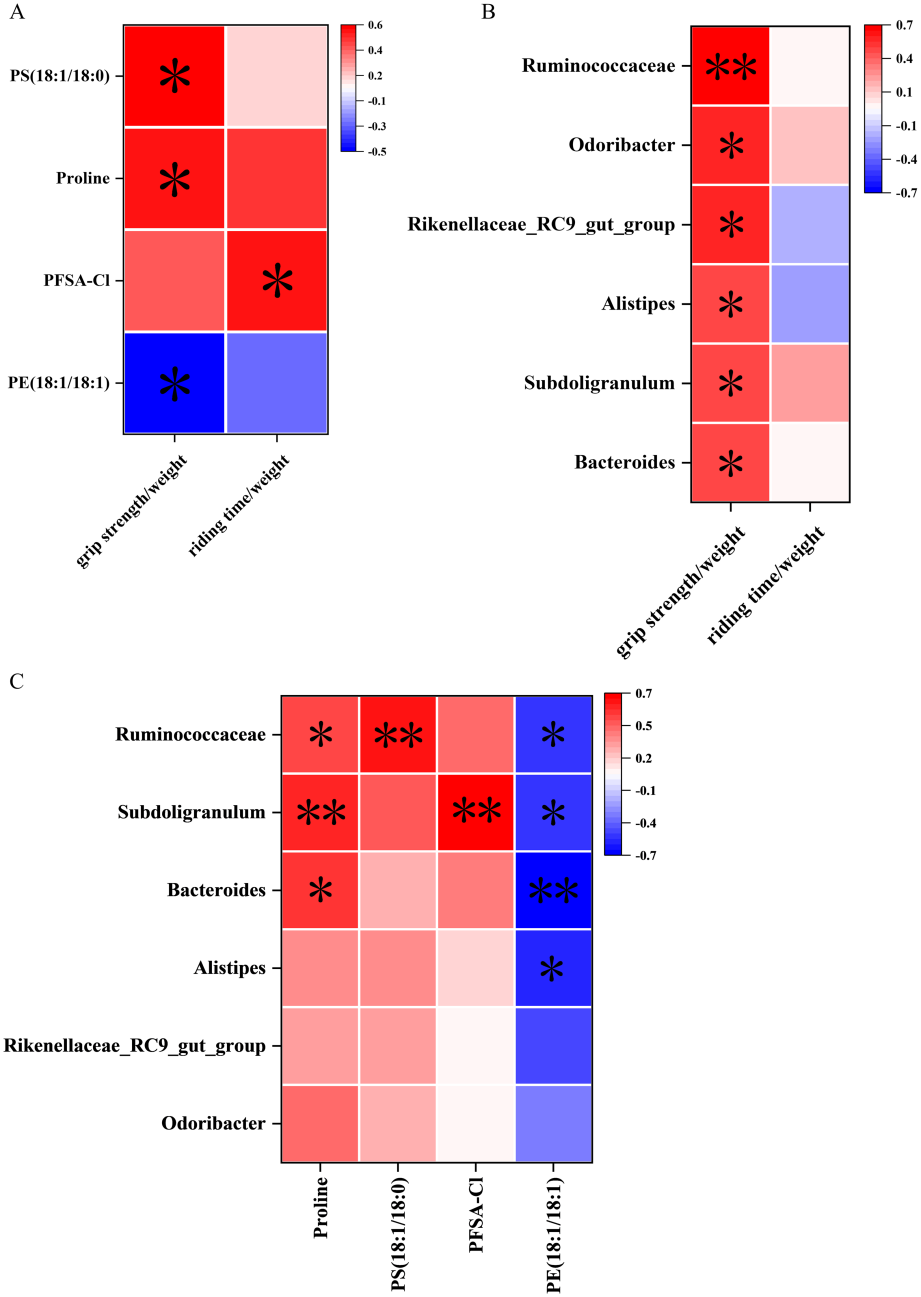
Correlation analysis of behavioral data, untargeted metabolomics, and 16S rRNA sequencing. **(A)** Correlation between cerebral cortex metabolites and behavioral data. **(B)** Correlation between gut microbiota data at the genus level and behavioral data. **(C)** Correlation between gut microbiota and metabolites at the genus level. *n* = 15; *p < 0.05, **p < 0.01.

Spearman’s correlation analysis was employed to elucidate the associations among behavioral data, differential metabolites, and genus-level differential gut microbiota. In the correlation analysis between behavioral data and differential metabolites, proline (*p* < 0.05), PS (18:1/18:0) (*p* < 0.05), and PFSA-CI (*p* < 0.05) exhibited significant positive correlations with the grip strength/weight or the riding time/weight, while PE (18:1/18:1) (*p* < 0.05) showed a significant negative correlation with the grip strength/weight (Figure 7A).

In the correlation analysis between behavioral data and genus-level differential gut microbiota, *Ruminococcaceae* (*p* < 0.01)*, Subdoligranulum* (*p* < 0.05)*, Bacteroides* (*p* < 0.05)*, Alistipes* (*p* < 0.05)*, Rikenellaceae_RC9_gut_group* (*p* < 0.05)*, and Odoribacter* (*p* < 0.05) were significantly positively correlated with the grip strength/weight (Figure 7B).

In the correlation analysis between differential metabolites and genus-level differential gut microbiota, proline showed significant positive correlations with *Ruminococcaceae* (*p* < 0.05)*, Subdoligranulum* (*p* < 0.01)*, and Bacteroides* (*p* < 0.05). Moreover, PS (18:1/18:0) showed significant positive correlations with *Ruminococcaceae* (*p* < 0.01). In addition, PFSA-CI showed significant positive correlations with *Subdoligranulum* (*p* < 0.01). However, PE (18:1/18:1) was significantly negatively correlated with *Ruminococcaceae* (*p* < 0.05)*, Subdoligranulum* (*p* < 0.05)*, Bacteroides* (*p* < 0.01)*, and Alistipes* (*p* < 0.05) (Figure 7C).

## 4 Discussion

**Figure 8:**
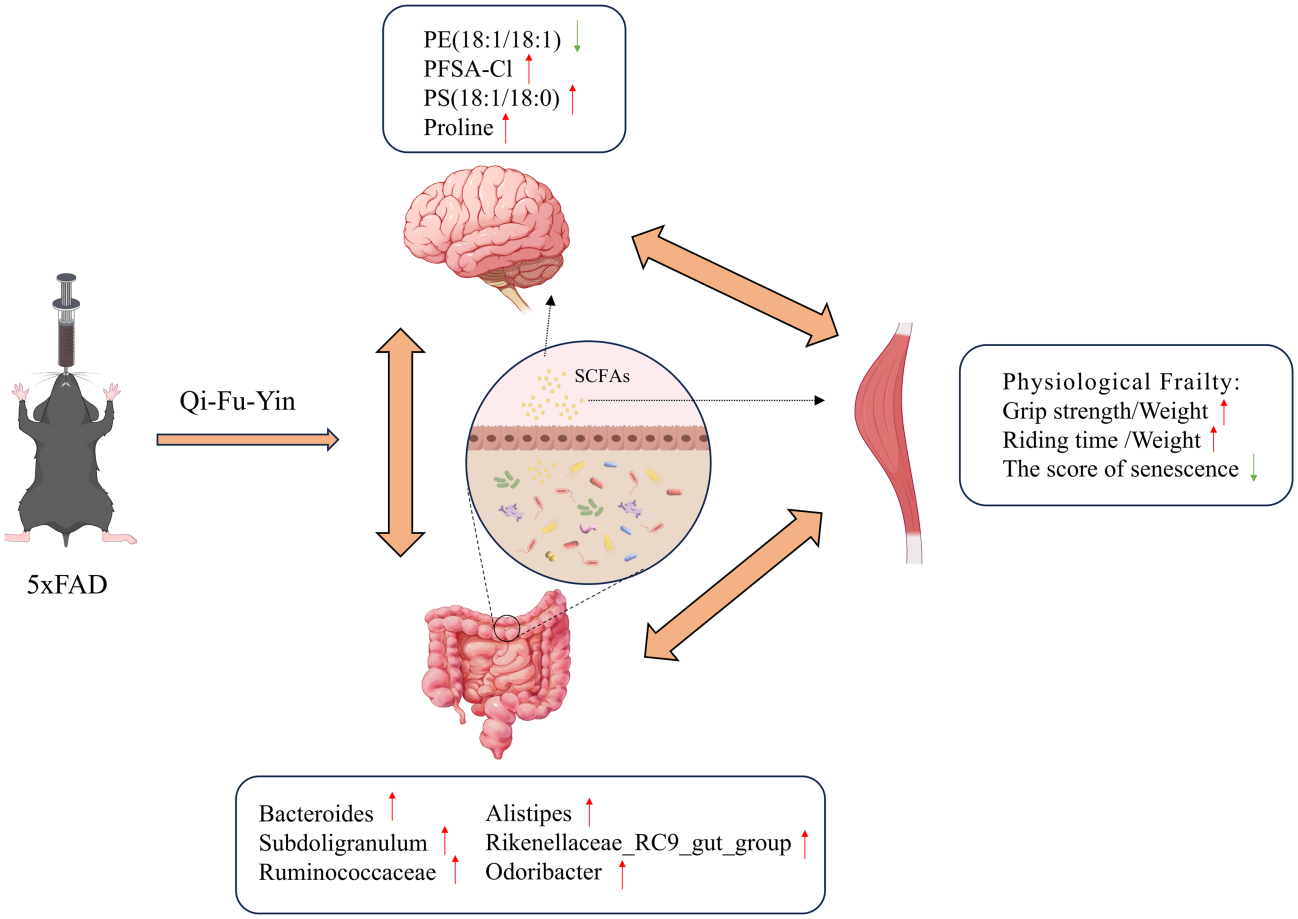
Qi-Fu-Yin ameliorates physiological frailty in male 5xFAD mice through remodeling the gut microbiota and modulating the cerebral cortex metabolism.

In this study, we mainly focused on physiological frailty in AD. Based on untargeted metabolomics and 16S rRNA gene sequencing analysis, we provided initial clues for the potential communication between the gut and the brain in the progression of physiological frailty in AD (Figure 8).

Previous studies (Duchowny et al., 2022; Adegoke et al., 2017) have shown that the motor function and coordination capability, which were evaluated by the grip strength test and rotarod test, were lower in the 5xFAD mice compared with the WT mice. Our study found that these indicators were higher in the 5xFAD mice with QFY intervention compared with the 5xFAD mice. The frailty score was lower in the 5xFAD mice with QFY intervention compared with the 5xFAD mice. Pathological analysis showed that the area of SA-β-gal, the area of amyloid deposition, and the area of co-located amyloid deposition and SA-β-gal were higher in 5xFAD mice than those in WT mice. These elevated indices could be lower in 5xFAD mice with QFY treatment. Additionally, preliminary observational evidence indicated higher lipofuscin content in 5xFAD mice compared to WT mice, while QFY treatment lowered this content. These results indicated that not only can QFY alleviate physiological frailty in male 5xFAD mice, but it can also delay brain aging that accompanies physiological frailty.

The technological innovations of various omics have become the most promising tools to investigate AD (Aerqin et al., 2022). In this study, we used LC–MS-based untargeted metabolomics. Biomarkers were screened by conducting multivariate statistical analyses of cerebral cortex metabolites. Several metabolites, including proline, PS (18:1/18:0), and PFSA-CI, were lower in the 5xFAD mice relative to the WT mice, while PE (18:1/18:1) was higher in the 5xFAD mice relative to the WT mice; these were all restored by the intervention of QFY. Studies have demonstrated that arginine and proline metabolism was shared between aging and AD, which held a crucial position in the transition process from healthy to mild cognitive impairment and ultimately to AD (Xie et al., 2021). Proline is vital in arginine and proline metabolism. It is an ideal osmotic regulator, a protective substance for membranes and enzymes, and a free radical scavenger (Lv et al., 2022). It induces mitophagy by activating AMP-activated protein kinase *α* and upregulating Parkin expression, enhancing mitochondrial clearance, and finally recovering cell metabolism (Choudhury et al., 2024). In humans, proline and glutamate form arginine *de novo*, and arginine is generally agreed to confer health benefits by stimulating the creation of NO, thus leading to an enhanced blood flow in the skeletal muscle fibers (Wu et al., 2021). PS (18:1/18:0) is one of the common types of phosphatidylserine. Phosphatidylserine, an important phospholipid in cell membranes, can enhance cognitive function and memory, protect the nervous system, regulate cell membrane functions (by maintaining its integrity and fluidity, and by influencing ion channels and receptors), and promote muscle recovery after exercise (Szondy et al., 2022; Kim et al., 2014). PFSA-CI is a kind of perfluoroalkyl sulfonic acid; detailed discussions and in-depth exploration of its function are notably rare in the existing literature. PE (18:1/18:1) is one of the common types of phosphatidylethanolamine. It is reported that an excess amount of phosphatidylethanolamine can increase the activity of β-secretase and *γ*-secretase, which promotes the activation of the amyloidogenic pathway, thus driving the production of Aβ (Calzada et al., 2016). These indicated that QFY treatment may improve male 5xFAD mice by regulating metabolites.

Changes in microbiota composition due to aging have been demonstrated to influence AD development (Kesika et al., 2021). In this study, 16S rRNA sequencing of fecal samples was conducted to investigate the alterations in microbiological composition after QFY intervention. The relative abundances of *Ruminococcaceae, Subdoligranulum, Bacteroides, Alistipes, Rikenellaceae_RC9_gut_group, and Odoribacter* were all significantly higher in the QFY group relative to the 5xFAD group. *Ruminococcaceae* depletion is associated with AD and age-related sarcopenia, and supplementation with beneficial microorganisms such as *Ruminococcaceae* may lead to better muscle function (Wang M. et al., 2024; Vogt et al., 2017). *Subdoligranulum* was associated with slower biological aging (Singh et al., 2024). Increased *Bacteroides*, *Alistipes*, and *Odoribacter* connect to both better cognitive performance and the attenuation of muscle injury after strenuous exhaustive exercise (Zhang S. et al., 2021; Liang et al., 2022; Zhang X. et al., 2021; Zhang et al., 2024). *Rikenellaceae_RC9_gut_group* is deemed a marker of healthy gut microbiota (Breyer et al., 2024). Notably, *Ruminococcaceae, Subdoligranulum, Bacteroides, Alistipes, Rikenellaceae_RC9_gut_group,* and *Odoribacter* are all putative SCFA-producing bacteria (Liang et al., 2022; Singh et al., 2022; Yang J. et al., 2023; Holman and Gzyl, 2019). Therefore, they can reduce the permeability of the blood–brain barrier through SCFAs (including acetate, propionate, and butyrate) and exert anti-neuroinflammatory and immunomodulatory effects (Xie et al., 2023). Some studies suggest that the specific mechanism is that SCFAs activate Treg cells mainly through binding to the Ffar2 receptor on Treg cells, which mediates a series of effects such as enhancing Treg cell proliferation and suppressive capacity, and also by inhibiting histone deacetylase, to increase histone acetylation and regulate gene expression related to Treg cell function, and ultimately rescue the progression of mild cognitive impairment (Yang J. et al., 2023; Smith et al., 2013). Moreover, new evidence indicates that the regulation of the microbiome to skeletal muscle is achieved partly by adjusting the metabolisms of SCFAs that might act directly on muscle or indirectly on the brain (Zhang et al., 2024; Daily and Park, 2022). It is not surprising then that gut microbiota and microbial metabolites (especially SCFAs) are also found to be involved in the development of other brain disorders such as Parkinson’s disease (Duan et al., 2024). In our study, butyrate metabolism was identified as an important metabolic pathway between the WT mice and the 5xFAD mice, as well as between the 5xFAD mice and the 5xFAD mice with QFY treatment. Butyrate can modulate macrophage polarization and the intestinal barrier function, eventually leading to the reduction of muscle atrophy (Liu et al., 2023). Dietary supplementation with butyrate can upregulate PGC1-*α*, subsequently promoting an increase in type I muscle fibers in mouse models (Henagan et al., 2015; Huang et al., 2017). Analogously, the acetate originating from the intestinal tract is essential for maintaining the quantity and power of skeletal muscles in mice (Kobayashi et al., 2024), and it benefits muscle energetics as exercise-associated post-biotics (Ismaeel et al., 2023). Our study found that *Ruminococcaceae, Subdoligranulum, Bacteroides, Alistipes, Rikenellaceae_RC9_gut_group, and Odoribacter* were all significantly positively correlated with the grip strength/weight.

However, this study has some limitations. First, to initially focus on a single-sex model for variable simplification and to clarify the core effect trend of QFY, only male mice were used in this experiment. This may limit the generalizability of QFY’s effects, and whether QFY exerts consistent or differential effects across sexes remains to be further investigated. Additionally, although we have made every effort to minimize experimental variability, the raw data of some metabolites showed relatively large standard errors compared with their means, and this part of the high variability may affect the robustness of some significant differences. Meanwhile, the associated mechanism between the gut microbiota and metabolites remains to be further verified, as does whether these findings can be generalized to human subjects complemented by *in vivo* imaging evidence.

## 5 Conclusion

The results of our study based on cerebral cortex metabolomics and 16S rRNA gene sequencing analyses demonstrate that QFY is an effective treatment for physiological frailty in male 5xFAD mice. The therapeutic functions of QFY may be attributable to its regulation of gut microbial structure, especially the abundances of bacterial genera *Ruminococcaceae, Subdoligranulum, Bacteroides, Alistipes, Rikenellaceae_RC9_gut_group, and Odoribacter*. Furthermore, the restoration of metabolites such as proline, PS (18:1/18:0), PFSA-CI, and PE (18:1/18:1) may also play a part in the mechanism of action of QFY. Overall, the findings preliminarily demonstrated that physiological frailty in male 5xFAD mice is associated with metabolic disorders and microbial dysbiosis, and QFY exerts protective effects by acting on the muscle-gut-brain axis.

## Data Availability

The raw data supporting the conclusions of this article will be made available by the authors, without undue reservation.

## Footnotes

1
http://cnij.imjoy.io/

2
http://www.metaboanalyst.ca/

3
http://www.kegg.jp

4
https://docs.qiime2.org/2019.4/tutorials/
