# Supplementary material for: Qi-Fu-Yin ameliorates physiological frailty in male 5xFAD mice through remodeling the gut microbiota and modulating the cerebral cortex metabolism
Source: Front Aging Neurosci. 2025 Dec 16;17:1622286. doi: 10.3389/fnagi.2025.1622286 (PMC12750344; doi:10.3389/fnagi.2025.1622286)
Supplement: Supplementary file 1 [file Table_1.docx]

| Degree of frailty | | | | | | | |
| --- | --- | --- | --- | --- | --- | --- | --- |
| Aging Score Indicators | | | 1 Point | 2 Points | 3 Points | 4 Points | 5 Points |
| 1.  Behavioral Responses | 1.1  Responsiveness | Responds within 30 seconds in most behavioral tests | Normal behavior | A. Abnormal behavior, but no decrease in agility | Significant decrease in agility | No active movement except when in physical contact | Immobile |
|  |  |  |  | B. Restlessness |  |  |  |
|  | 1.2  Passive Avoidance Response | Makes avoidance response when neck skin is pinched or forelimbs are restrained | Normal avoidance response | Decreased avoidance response when neck skin is pinched | Loss of avoidance response when neck skin is pinched, but maintains normal response when flipped over | Loss of avoidance response when neck skin is pinched, but normal response when forelimbs are restrained | Loss of avoidance response |
|  | 1.3  Mental State Index | Number of movements of mice per unit time |  | More than 30 times per minute, flexible response to external stimuli | 10-30 times per minute, relatively flexible response to external stimuli | Less than 10 times per minute, relatively slow response to external stimuli | Less than 5 times per minute, slow response to external stimuli |
| 2.  Skin and Hair | 2.1  Luster | Hair is smooth and shiny | Smooth hair with average luster | Low luster | Loss of luster | Loss of luster, hair is obviously dirty | Loss of luster, hair is obviously very dirty |
|  | 2.2  Roughness | Number of tangles in hair that can be touched on the head, neck, and back | No tangles | Tangled area is smaller than the area of the head | Tangled area is smaller than twice the area of the head | Tangled area is smaller than three times the area of the head | Tangled area is larger than twice the area of the head |
|  | 2.3  Density | Degree of hair loss or sparseness on the head, neck, and back (excluding those caused by skin ulcers or periocular lesions) | No hair loss or sparseness | A. Hair loss area is smaller than the area of the mouse's head | A. Hair loss area is larger than the area of the mouse's head or smaller than 1/4 of the mouse's total body area  B. Sparse area is larger than half of the mouse's total body area | Hair loss area is larger than 1/4 but smaller than 1/2 of the mouse's total body area | Hair loss area is larger than 1/2 of the mouse's total body area |
|  |  |  |  | B. Sparse area is smaller than half of the mouse's total body area |  |  |  |
|  | 2.4  Skin Ulcers | Skin ulcers and healed ulcers | No ulcers | Ulcers healed or scabbed | Ulcer area is smaller than the area of the mouse's head | Ulcer area is larger than the area of the mouse's head but smaller than 1/4 of the mouse's total body area | Ulcer area is larger than 1/4 of the mouse's total body area |
| 3.  Eyes | 3.1  Periocular Lesions | Ocular catarrhal lesions (red bloodshot eyes) or eyelid swelling | No lesion | Periocular catarrhal lesions | Periocular catarrhal lesions extend to the nose | Periocular catarrhal lesions extend beyond the nose |  |
|  | 3.2  Corneal Opacity | Directly observable corneal opacity and decreased corneal transparency | No opacity | Iris opacity | Obvious iris opacity | Complete corneal opacity |  |
|  | 3.3  Corneal Ulcers | Directly observable corneal ulcers | No ulcers | Ulcers at the palpebral fissure | Increased number of ulcer sites | Complete corneal ulceration |  |
| 4.  Spine | 4.1  Kyphosis | Directly observable or palpable kyphosis | Normal spinal curvature | Increased spinal curvature, disappears when pressed with fingers | Increased spinal curvature, disappears when combined with finger pressing and pulling |  | Permanent kyphosis |
|  | 4.2  Degree of Spinal Curvature |  | No spinal curvature | Spinal curvature: 150° < θ < 180° | Spinal curvature: 120° < θ < 150° | Spinal curvature: 90° < θ < 120° | Spinal curvature: θ < 90° |

**Supplementary Table 1** Degree of Frailty Scoring Table
