# Supplementary material for: Qi-Fu-Yin ameliorates physiological frailty in male 5xFAD mice through remodeling the gut microbiota and modulating the cerebral cortex metabolism
Source: Front Aging Neurosci. 2025 Dec 16;17:1622286. doi: 10.3389/fnagi.2025.1622286 (PMC12750344; doi:10.3389/fnagi.2025.1622286)
Supplement: Supplementary file 2 [file Table_2.docx]

Supplementary Material

**Supplementary Table 2** Metabolites changes in 5xFAD mice

| Metabolite name | Mean±SEM | | | PLS-DA.VIP | 5xFAD *vs.* 5xFAD+QFY | WT *vs.* 5xFAD |
| --- | --- | --- | --- | --- | --- | --- |
|  | WT | 5xFAD | 5xFAD+QFY |  |  |  |
| PG(16:0/22:4) | 12576326±2100200 | 33995399±6658009 | 8773556±1676850 | 1.92 | ↑** | ↓* |
| PG(20:4/16:0) | 10333404±2013362 | 12821604±1235862 | 6144104±805753 | 2.13 | ↑** | ↓ |
| PC(17:0/14:1)-d5 | 101794067±29602236 | 108305965±16915273 | 53526628±15645132 | 1.59 | ↑* | ↓ |
| PS(18:1/18:0) | 24357641±5908524 | 23496530±5318272 | 45078254±6946043 | 1.68 | ↓* | ↑ |
| PG(22:6/16:0) | 12824751±1250950 | 3956344±593391 | 6005580±498680 | 1.69 | ↓* | ↑** |
| [1-[(2-amino-3-oxobutoxy)-hydroxyphosphoryl]oxy-3-heptadecanoyloxypropan-2-yl] icosa-4,7,10,13-tetraenoate | 4669642±1054939 | 9970082±1164616 | 6715293±531825 | 1.62 | ↑* | ↓* |
| PC(15:0/22:6) | 38576600±14489764 | 7363530±1406790 | 50988359±11042144 | 2.12 | ↓** | ↑ |
| PG(22:6/22:6) | 44540776±4026026 | 41955681±4603792 | 92086764±12287803 | 2.09 | ↓** | ↑ |
| PI(16:0/22:6) | 14946402±3261761 | 265394±64368 | 1061792±52863 | 2.46 | ↓** | ↑* |
| PI(18:1/20:4) | 8294399±3684999 | 16105951±2484459 | 4512525±499251 | 2.09 | ↑** | ↓ |
| PI(18:0/22:6) | 5650926±1375479 | 10041629±1878363 | 4546525±1045794 | 1.63 | ↑* | ↓ |
| N-Acetylisoleucine | 28346514±1348739 | 32832168±2343580 | 25448265±1554123 | 1.65 | ↑* | ↓ |
| o-Cresol sulfate | 1756558±1325831 | 2992870±784941 | 435865±122736 | 1.81 | ↑* | ↓ |
| Gluconic acid | 31305223±2495124 | 25766342±1110168 | 39882924±3225515 | 2.14 | ↓** | ↑ |
| Camalexin | 18715825±4929783 | 17325163±6876206 | 97924312±10526558 | 2.36 | ↓** | ↑ |
| Daucic acid | 75653066±7393614 | 102561969±12631354 | 68052197±5552319 | 1.63 | ↑* | ↓ |
| sn-Glycerol-3-phosphoethanolamine | 238474356±29924033 | 212769370±20808240 | 310821634±23635901 | 1.87 | ↓* | ↑ |
| 7-Epi-12-hydroxyjasmonic acid | 727423±118388 | 1191958±172854 | 685841±120182 | 1.58 | ↑* | ↓ |
| PFAP-diPAP | 29135635±2589801 | 31472230±2573535 | 22895390±1882962 | 1.72 | ↑* | ↓ |
| 3,5-Dihydroxycinnamic acid sulfate | 93201±65005 | 30775±14084 | 392687±92367 | 2.11 | ↓** | ↑ |
| Talaroflavone | 55547994±2382205 | 64336832±5096447 | 50214236±2427958 | 1.61 | ↑* | ↓ |
| 2-Furancarboximidamide | 21841007±2719130 | 29434976±780860 | 22123762±2395447 | 1.87 | ↑* | ↓* |
| PFSA-Cl | 120564069±11881092 | 109439235±11477190 | 146618054±9129547 | 1.69 | ↓* | ↑ |
| PFSM-ammonio | 396314144±27407015 | 475926443±44981950 | 316379205±34476659 | 1.75 | ↑* | ↓ |
| Platyphyllene | 293056±56489 | 110173±50422 | 347742±97843 | 1.56 | ↓* | ↑* |
| Maleic acid | 355001200±36424460 | 368244293±41236817 | 225308978±29708682 | 1.74 | ↑* | ↓ |
| (4E)-7-Acetoxy-6-hydroxy-2-methyl-10-oxo-3,6,7,8,9,10-hexahydro-2H-oxecin-3-yl (2E)-2-butenoate | 36068415±3425877 | 34832906±2491879 | 44742351±3097623 | 1.67 | ↓* | ↑ |
| N-((1E,3E)-4-(2H-benzo[3,4-d]1,3-dioxolan-5-yl)-1-azabuta-1,3-dienyl)-4-pyridy lcarboxamide | 32361477±6458149 | 34946423±3210610 | 19301766±4808602 | 1.76 | ↑* | ↓ |
| 8-bromo-6-chloro-3-(4-hydroxyphenyl)-4-methylchromen-2-one | 92706872±9664632 | 117317106±12323441 | 75051101±3903350 | 1.86 | ↑* | ↓ |
| PFSA-ether | 21060032±2990616 | 28513892±4521870 | 16325619±1419808 | 1.65 | ↑* | ↓ |
| Pyrroline hydroxycarboxylate | 1464669540±149939563 | 1132487281±79518558 | 1509441043±107528845 | 1.78 | ↓* | ↑ |
| LPG(20:4) | 23977296±3818172 | 47036474±7016182 | 11727046±2905679 | 2.12 | ↑** | ↓* |
| 6-Benzyl-3-butan-2-yl-9-(7-hydroxy-6-oxooctyl)-1,4,7,10-tetrazabicyclo[10.4.0]hexadecane-2,5,8,11-tetrone | 9011±1824 | 20098±4093 | 6065±3458 | 1.69 | ↑* | ↓* |
| 3-O-beta-(6''-trans-caffeoyl)-galactopyranosyl quercetin | 18349257±5295091 | 19041834±3380427 | 9392165±982288 | 1.66 | ↑* | ↓ |
| PG(16:0/16:0) | 5913798±1204297 | 15761477±1530300 | 7305365±1088663 | 2.15 | ↑** | ↓** |
| PG(18:1/16:0) | 122005006±20660448 | 161208140±23264009 | 81715820±7260491 | 1.89 | ↑* | ↓ |
| 4-Hydroxybenzotriazole | 538066631±31326833 | 580502614±45406061 | 436031909±22644997 | 1.76 | ↑* | ↓ |
| PE(18:1/18:1) | 116196728±3418754 | 116564005±6229679 | 91311306±5400682 | 1.83 | ↑* | ↓ |
| Camphene | 139421265±18214905 | 185010898±35791237 | 92033658±12153407 | 1.56 | ↑* | ↓ |
| 6-Methyl-2-pyridinecarboxylic acid | 49275387±5663496 | 47410769±5422722 | 65387180±5487384 | 1.6 | ↓* | ↑ |
| PC(16:0/18:2) | 251547759±42660079 | 227260524±42848945 | 491604311±102407848 | 1.69 | ↓* | ↑ |
| PE(20:3/16:0) | 341169797±102982798 | 55704888±18177040 | 157688491±36700046 | 1.7 | ↓* | ↑* |
| PE(16:0/22:6) | 17229053±7890402 | 3632295±1313946 | 11744132±1724167 | 2.04 | ↓** | ↑ |
| PC(18:0/18:2) | 268287368±17541113 | 623026945±46453578 | 487473390±28784022 | 1.6 | ↑* | ↓** |
| PC(17:0/18:1)-D5 | 45675697±2084813 | 45975451±3077933 | 24433674±1197866 | 2.32 | ↑** | ↓ |
| 5,6,7,8-Tetrahydro-4-methylquinoline | 26061732±1337436 | 31301054±2253947 | 24329644±1781772 | 1.59 | ↑* | ↓ |
| Creatine | 884365605±49149012 | 949368470±56651239 | 727863932±36525120 | 1.89 | ↑* | ↓ |
| [1-[21-acetamido-18-benzyl-3-(1-methoxyethyl)-4,9,10,12,16-pentamethyl-15-methylidene-2,5,8,11,14,17,20-heptaoxo-22-propan-2-yl-1,19-dioxa-4,7,10,13,16-pentazacyclodocos-6-yl]-2-methylpropyl] 2-acetamido-3-hydroxy-4-methylpentanoate | 8122946±908692 | 5416907±1071509 | 10689853±753324 | 2.04 | ↓** | ↑ |
| Methionine sulfoxide | 305462619±36261929 | 390228830±31480790 | 256161708±36424989 | 1.79 | ↑* | ↓ |
| 2-Methylbutylamine | 8637537±860552 | 9229532±519678 | 5902256±231178 | 2.26 | ↑** | ↓ |
| Furoylglycine | 61129406±10922169 | 70568529±8481174 | 39409355±6826217 | 1.77 | ↑* | ↓ |
| 5-[(1-Iminoethyl)amino]-2-aminopentanoic acid | 1493861±132838 | 2760982±268405 | 1316990±232465 | 2.07 | ↑** | ↓** |
| Isovalerylglutamic acid | 20857008±3678982 | 25203898±6333494 | 7403937±3270492 | 1.69 | ↑* | ↓ |
| Pivaloylcarnitine | 418521793±44977795 | 407202668±45002889 | 584218260±28787992 | 1.9 | ↓* | ↑ |
| Dimethyl sulfoxide | 946828835±124321312 | 1322238380±148674867 | 687207553±86693893 | 1.97 | ↓** | ↑ |
| Dibutyl phthalate | 492663918±33633617 | 513822691±31352111 | 399245036±31412627 | 1.69 | ↑* | ↓ |
| Oleamide | 17507919±8917733 | 17813933±2039931 | 4196187±1988831 | 2.18 | ↑** | ↓ |
| S-Adenosylhomocysteine | 76174071±14480696 | 50221564±8717007 | 89287300±6765682 | 1.94 | ↓** | ↑ |
| Proline | 961011505±24058492 | 944212016±26504894 | 1065047686±45912008 | 1.61 | ↓* | ↑ |
| 3-Amino-2,2-dimethylpropanoic acid | 1080820727±50593133 | 864255093±52364085 | 1708730173±136142656 | 2.32 | ↓** | ↑* |

n = 5-6. ↑: up-regulated, ↓: down-regulated;**P*<0.05, ***P*<0.01. *vs* 5xFAD mice, Statistical differences are assessed by the Student’s *t*-test.
